# Synthesis and characterization of semisynthetic analogs of the antifungal occidiofungin

**DOI:** 10.3389/fmicb.2022.1056453

**Published:** 2022-12-13

**Authors:** Mengxin Geng, Nopakorn Hansanant, Shi-En Lu, Steve W. Lockless, Ronald Shin, Ravi Orugunty, Leif Smith

**Affiliations:** ^1^Department of Biology, Texas A&M University, College Station, TX, United States; ^2^Sano Chemicals Inc., Bryan, TX, United States; ^3^Department of Biochemistry, Molecular Biology, Entomology and Plant Pathology, Mississippi State University, Mississippi State, MS, United States; ^4^Central Alabama High-Field NMR Facility, Structural Biology Shared Facility, Cancer Center University of Alabama at Birmingham, Birmingham, AL, United States

**Keywords:** antifungal, *Candida*, NMR, semisynthetic analog, NRPS, occidiofungin

## Abstract

Occidiofungin is a broad-spectrum antifungal compound produced by *Burkholderia contaminans* MS14. It is a cyclic glycol-lipopeptide with a novel beta-amino acid (NAA2) containing a hydroxylated C18 fatty acid chain with a xylose sugar. This study reports a strategy to produce semisynthetic analogs of occidiofungin to further explore the structure activity relationships of this class of compounds. Oxidative cleavage of the diol present on carbons five C(5) and six C(6) removes the xylose and twelve carbons of the fatty acid chain. The resulting cyclic peptide product, occidiofungin aldehyde, is devoid of antifungal activity. However, the free aldehyde group on this product can be subjected to reductive amination reactions to provide interesting semisynthetic analogs. This chemistry allows the quick generation of analogs to study the structure activity relationships of this class of compounds. Despite restoring the length of the aliphatic side chain by reductive amination addition with undecylamine or dodecylamine to the free aldehyde group, the obtained analogs did not demonstrate any antifungal activity. The antifungal activity was partially restored by the addition of a DL-dihydrosphingosine. The dodecylamine analog was demonstrated to still bind to the cellular target actin, suggesting that the diol on the side chain of native occidiofungin is important for entry into the cell enabling access to cellular target F-actin. These results show that the alkyl side chain on NAA2 along with the diol present on this side chain is important for occidiofungin’s antifungal activity.

## Introduction

The emergence of pathogenic fungi resistant to currently used antifungals is a severe clinical problem (Candida auris Clinical Update—September, 2017). *Candida* species, an important cause of infection and mortality in all hospitalized patients, have been reported to have gained resistance to clinically used azoles and echinocandins (Perlin, 2007; Whaley et al., 2017). Further, the emergence of multidrug resistant strains of *Candida auris* has added to the uncertainty of current antifungal treatment options being effective in the near future (Jeffery-Smith et al., 2018). In addition to the widespread resistance and limited spectrum of activity, current antifungal treatments lead to abnormal liver and kidney function tests (Ashley et al., 2006; Lewis, 2011). For instance, the adverse effects of ketoconazole are severe enough that FDA limits the oral use of this drug for the treatment of endemic mycoses and has restricted its use in cases when alternative antifungal therapies are not available or are not tolerated (FDA Drug Safety Communication, 2013). The use of ketoconazole is banned in Europe (European Medicines Agency, 2013). Therefore, it is reasonable to declare that the current state of antifungal therapies is limited by their safety and efficacy. In light of the various challenges in antifungal therapies, there is an urgent need to develop newer antifungals with less toxicity, novel mechanisms of action and improved spectrum of activity.

Occidiofungin is a novel antifungal compound isolated from *Burkholderia contaminans* MS14 (Lu et al., 2009) and its structure has been determined by nuclear magnetic resonance (Lu et al., 2009; Gu et al., 2011). Occidiofungin has a broad spectrum of activity against fungi with minimum inhibitory concentrations (MICs) in the low micromolar to nanomolar range (Ravichandran et al., 2019). Further, occidiofungin has been demonstrated to be efficacious in treating vulvovaginal candidiasis (VVC) in a mouse model (Ravichandran et al., 2019) and has been shown to be well tolerated by mice in toxicity studies (Tan et al., 2012). VVC is rapidly developing resistance to currently available therapeutics, and an established treatment method for the disease is not currently available (Zhang et al., 2014; Matheson and Mazza, 2017). Occidiofungin has a novel mechanism of action that is vastly different from other common classes of antifungals (Emrick et al., 2013; Ravichandran et al., 2019) The primary cellular target of occidiofungin is actin. Actin-mediated cellular processes in yeast, such as endocytosis, nuclear segregation, and hyphal formation, were all disrupted following addition of subinhibitory concentrations of occidiofungin (Ravichandran et al., 2019). Occidiofungin’s binding to actin interferes with F-actin filament cable stability and does not interfere with polymerization or depolymerization of actin filaments. Occidiofungin binds to F-actin with an estimated dissociation constant (Kd) of 1,050 nM and has a high saturation of binding, with a ratio of more than 20 molecules of occidiofungin to one actin monomer (Ravichandran et al., 2019) Given the high binding ratio, it was predicted that the microscopic Kd value (which captures the affinity of one occidiofungin molecule binding to actin) is significantly lower than the 1,050 nM dissociation constant.

**Figure 1:**
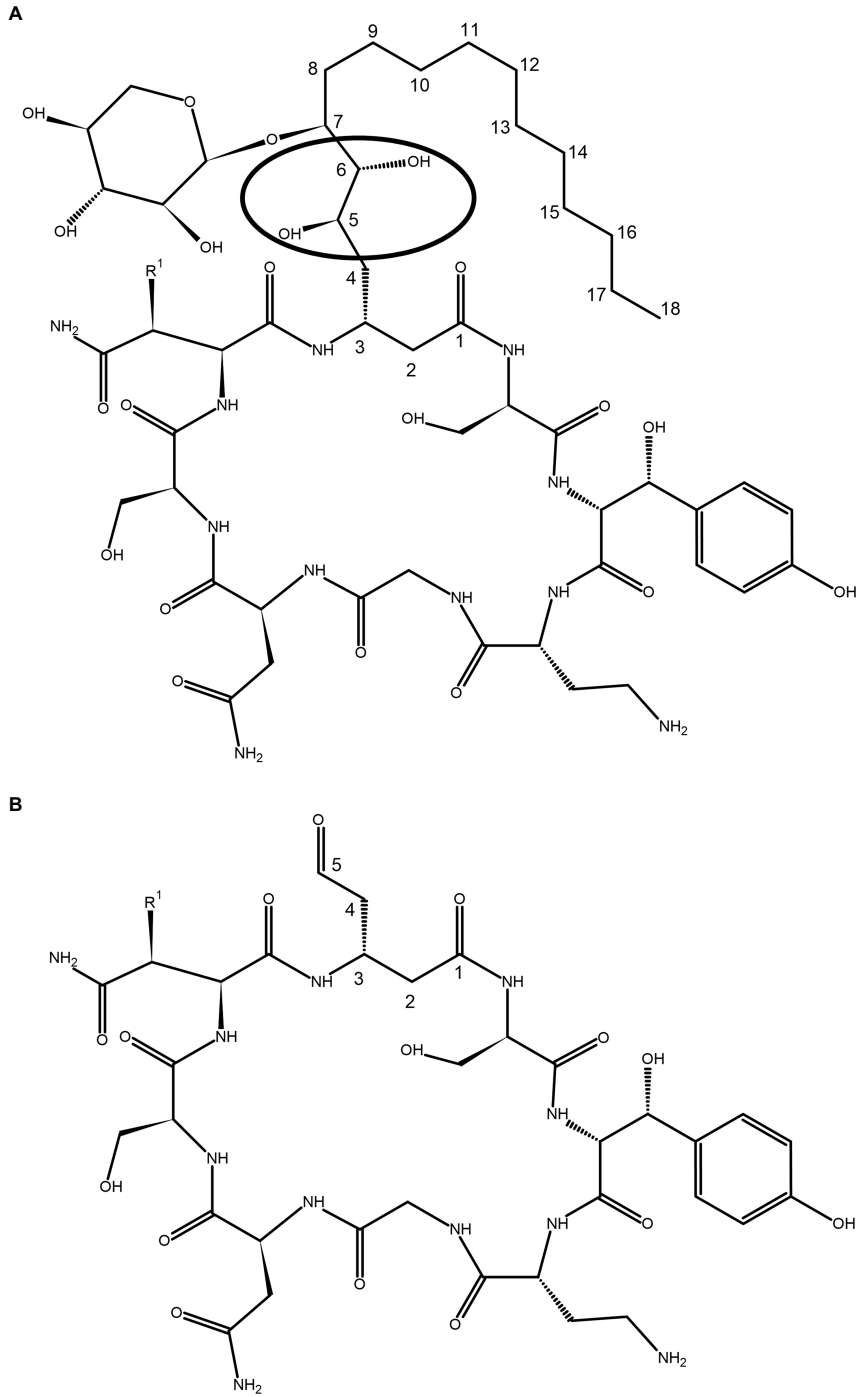
Covalent structure of **(A)** occidiofungin and **(B)** aldehyde analog. The circle indicates the position of the diol group where the oxidative cleavage occurs. The carbons in the novel amino acid (NAA) are numbered based on the carbonyl group of NAA labeled as carbon one C (1). R1 is a proton or hydroxyl group, representing the natural occurrence of an asparagine and beta hydroxy-asparagine variants of occidiofungin.

Occidiofungin is a novel cyclic peptide composed of eight amino acids. The amino acid at the second position (NAA2) is a novel beta-amino acid (3-amino-5,6,7-trihydroxy stearic acid) containing a xylose sugar. The hydroxy group on the seventh carbon C(7) of this amino acid is attached to a xylose moiety via a glycosidic linkage (Figure 1A). Very little is known about the structure activity relationship (SAR) of this structurally complex molecule. A xylose free analog of occidiofungin was produced from a xylose transferase mutant strain of MS14 named MS14KC1 (Chen et al., 2013). The yield of this product was 7-fold lower compared to native occidiofungin, suggesting that the presence of the xylose may be important for regulation or transport of occidiofungin from the bacterium. However, the isolated product did demonstrate that the xylose on the side chain is not important for the potent antifungal activity. The product had the same inhibitory activity against several strains of yeast as the native compound (Chen et al., 2013). Repeat-dose toxicity studies with occidiofungin shows a mild inflammatory response in murine models (Tan et al., 2012). This may be due to the presence of the xylose sugar which is not found in mammals. Therefore, a xylose-free analog of occidiofungin may be developed as a superior therapeutic option compared to native occidiofungin.

**Figure 2:**
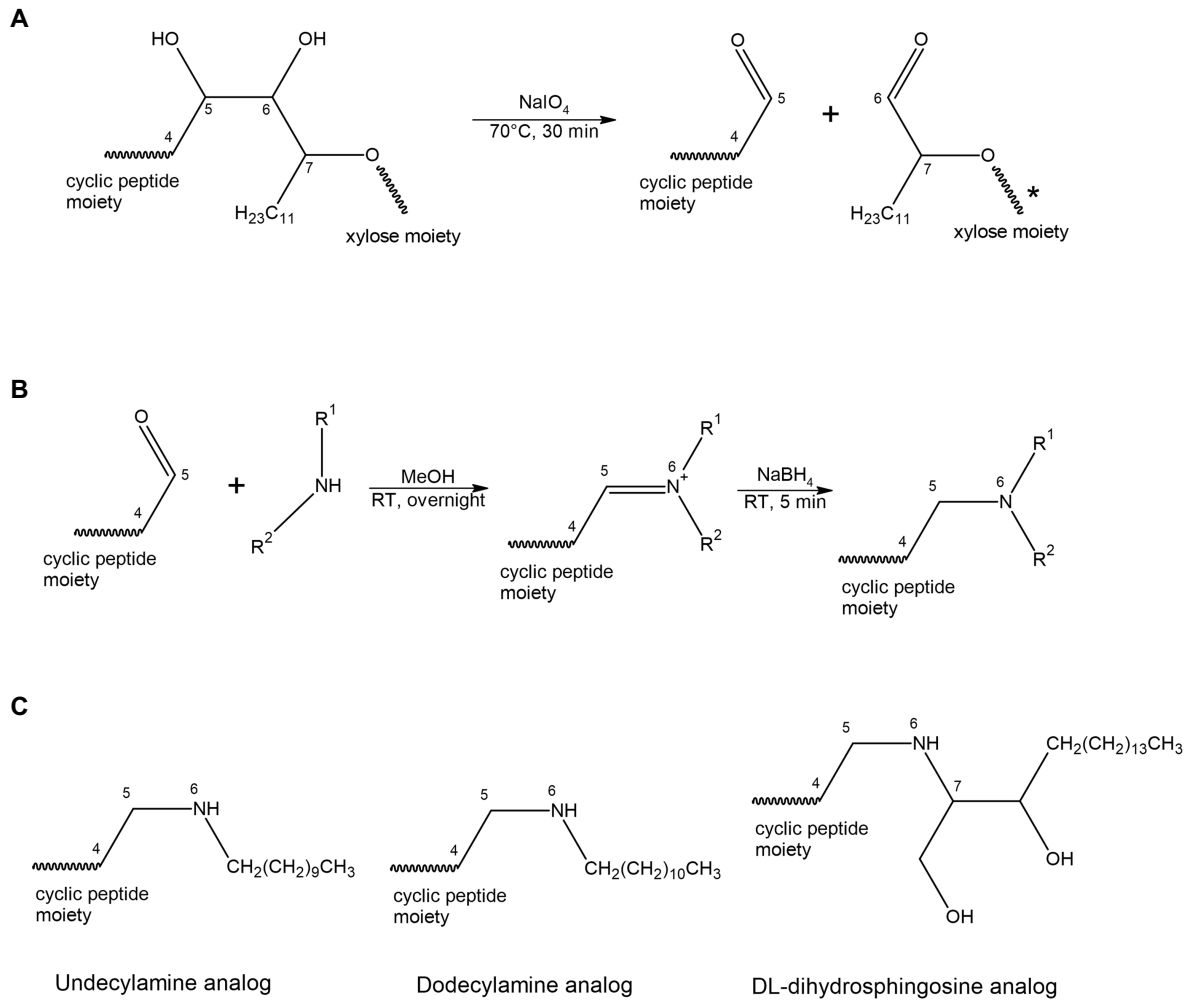
Scheme for oxidation **(A)** and reductive amination **(B)** reaction. **(A)** A periodate mediated oxidative cleavage of the vicinal diols removes the xylose and a portion of the aliphatic side chain on the NAA2. The reaction results in a cyclic peptide with a free aldehyde group. **(B)** A two-steps reductive amination procedure. The aldehyde group reacts with a primary amine to form an aldimine, followed by reduction with sodium borohydride to give the corresponding secondary amine. **(C)** Structural representation of the three acyl lipid analogs synthesized.

In this paper we disclose a semisynthetic route toward synthesizing novel analogs at the NAA2 position. A periodate mediated oxidation (Malaprade’s oxidation) was used to selectively remove the xylose and a portion of the aliphatic side chain on the NAA2 residue (Malaprade, 1934; Price et al., 1938; Figures 1, 2). The reaction affords a cyclic peptide with a free aldehyde that can be efficiently used in subsequent reductive amination reactions to introduce a variety of novel side chain analogs. As proof of principle, we took the aldehyde occidiofungin product and synthesized three analogs. We evaluated these analogs for their antifungal activity and their actin binding properties. The semisynthetic approach used to synthesize new NAA2 analogs can be used to expand the library of semisynthetic analogs of occidiofungin. This library will be useful for identifying semisynthetic analogs that may have novel clinical applications. Further, the study has promoted our understanding of the structure activity relationships of occidiofungin and promotes future studies at determining how the compound enters the cell. These findings may also be applied to promoting cellular uptake of other large molecules.

## Materials and methods

### Materials

Occidiofungin and the xylose free analog of occidiofungin were purified from culture broth of *B. contaminans* MS14 and the xylose transferase mutant strain of MS14 named MS14KC1, respectively, as previously described (Lu et al., 2009; Chen et al., 2013). Purified rabbit skeletal muscle filamentous actin (AKF99) was purchased from Cytoskeleton Inc. (Denver, CO). Sodium periodate (311448-5G), sodium borohydride (452882), undecylamine (94200-10ML), dodecylamine (325163-5ML), and DL-dihydrosphingosine (D6783-10MG) were purchased from Sigma (St. Louis, MO).

### Synthesis of semisynthetic analogs

Occidiofungin was dissolved in 50% acetonitrile (ACN) with 0.1% trifluoroacetic acid (TFA) at a concentration of 1 mg/ml. Sodium periodate was dissolved in ddH_2_O at a concentration of 1 mg/ml. Equal volumes of the two solutions were mixed thoroughly in a 10 ml centrifuge tube and was incubated at 70°C for 30 min. One milliliter of the reaction mixture was loaded on a BioRad Duoflow chromatography system with an analytical C18 column (Agilent® ZORBAX, Agilent Technologies, Santa Clara, *CA.* ODS, C18, 5 μm, 4.6 × 250 mm). The reaction mixture was separated as previously described with an isocratic flow of 20% MeOH in water (Li et al., 2015) and the resulting aldehyde analog of occidiofungin eluted at approximately 9 min. The desired product was freeze-dried and weighed on an analytical balance (Adventurer™ Pro AV114C, Ohaus Corporation, USA).

A two-step procedure of reductive amination was used to introduce a primary or secondary amine onto the aldehyde analog. The first step of reaction involves the formation of an intermediate carbinol amine, which is then dehydrated and protonated to form an iminium ion. Subsequent reduction of this iminium ion with sodium borohydride produces an alkylated amine product. The aldehyde analog of occidiofungin was dissolved in DMSO at a concentration of 10 mg/ml. Three amines (undecylamine, dodecylamine, and DL-dihydrosphingosine) were used for reductive amination. Aldehyde analog (10 μl of DMSO stock solution; 100 μg) was mixed with 10-fold excess of an amino lipid (molar ratio) in 400 μl pure methanol. The sample was then incubated at room temperature for at least 16 h. Solid sodium borohydride (6 mg) was weighted on an analytical balance (Adventurer™ Pro AV114C, Ohaus Corporation, USA) and was directly transferred into the reaction mixture, mixed well, and was incubated at room temperature for 5 min. The reaction mixture was diluted to 1 ml with 50% ACN 0.1% TFA and was separated by HPLC using a 30-min gradient from 90 to 20% water/ACN on the analytical C18 column. HPLC solvents contained 0.1% TFA. Products that eluted between 50% and 30% water were collected and analyzed by electrospray ionization mass spectrometry (ESI-MS) on a ThermoFisher DecaXP ion trap mass spectrometer. The yield of each HPLC sample was quantified by comparing the peak area of each analog to the peak area of a 100 μg standard of occidiofungin. Electrospray ionization mass spectrometry (ESI-MS) experiments were performed using a Thermo Scientific Q Exactive Focus. Sample was loop injected (10 μl) and acetonitrile with 0.1% formic acid was used as a mobile phase at a flow rate of 600 μl/min. The Q Exactive Focus HESI source was operated in full MS in positive mode. The mass resolution was tuned to 70,000 FWHM at m/z 200. The spray voltage was set to 3.5 kV, and the sheath gas and auxiliary gas flow rates were set to 40 and 10 arbitrary units, respectively. The transfer capillary temperature was held at 300°C and the S-Lens RF level was set at 50 v. Exactive Series 2.11 /Xcalibur 4.2.47 software was used for data acquisition and processing. The alkyne occidiofungin (alkyne-OCF) used for fluorescent microscopy was synthesized as previously described (Ravichandran et al., 2019).

### Nuclear magnetic resonance spectroscopy

NMR analysis of aldehyde occidiofungin analog was performed on a 5 mM sample dissolved in dimethyl sulfoxide (DMSO)-d6. The NMR data were collected on an Avance III HD-600 with a TCI Cryoprobe and an Avance III HD-850 with a TCI Cryoprobe. The ^1^H resonances were assigned according to standard methods (Wüthrich, 1986) using COSY, TOCSY, NOESY, and ^13^C-HSQC experiments (Bodenhausen and Ruben, 1980; Kumar et al., 1980; Braunschweiler and Ernst, 1983). The 1H-NMR, TOCSY, NOESY, COSY, and ^13^C-HSQC experiments were collected at 25°C. ^1^H chemical shifts were referenced to DMSO peak at 2.5 ppm. The TOCSY experiment was acquired with a 60 ms mixing time using the Bruker DIPSI-2 spinlock sequence. The NOESY experiment was acquired with a 400 ms mixing time. Phase sensitive indirect detection for NOESY, TOCSY, and COSY experiments was achieved using the standard Bruker pulse sequences. Peaks were assigned using NMRView (Johnsona and Blevinsb, 1994).

### Minimum inhibitory concentration assays

The minimal inhibitory concentration (MIC) is the lowest concentration of compound that inhibits the visible growth of the yeast after 24 h of incubation. MIC assays were performed in duplicate following a modified version of the CLSI M27-A3 protocol as previously described (Ravichandran et al., 2019).

### Fluorescent microscopy and competitive binding assay

Fluorescent microscopy and competitive binding assays were performed as previously described (Ravichandran et al., 2019) Briefly, *Saccharomyces cerevisiae* cells at OD_600_ of 0.6 to 0.8 was incubated with 1× MIC of alkyne-occidiofungin at 30°C. After 30 min, the cells were fixed then permeabilized. Click reaction was performed with azide-derivatized Alexa Fluor 488 as per the kit-supplied protocol (Click-iT EdU imaging kit; Thermo Fisher Scientific). The cells were suspended in VECTASHIELD® Antifade Mounting Medium and visualized with a Leica DM 6B microscope. Competitive assays were performed by incubating the *S. cerevisiae* cells with the dodecylamine analog or native occidiofungin for 5 min, prior to the addition of 1X MIC of alkyne-occidiofungin for 30 min. Pretreatment with native occidiofungin or the dodecylamine analog was performed with concentrations of 1X MIC of native occidiofungin concentration. The cells were then fixed, permeabilized and Click reaction was performed as previously described (Ravichandran et al., 2019).

### Actin co-sedimentation assay

Actin binding experiments were performed as previously described for native occidiofungin (Ravichandran et al., 2019). An Agilent 1,200 front end chromatography system and a TSQ Quantum™ Access Triple Quadrupole Mass Spectrometer was used to analyze phalloidin, native occidiofungin, aldehyde analog, xylose free occidiofungin, dodecylamine analog, and the DL-dihydrosphingosine analog binding to F-actin. Following a 10 μl injection, samples containing dodecylamine analog of occidiofungin were separated using a 15-min water/ACN (containing 0.2% formic acid) gradient starting from 95 to 40% water on a C18 column (SinoChrom ODS-BP 5 μm, 2.1 mm × 50 mm). Samples containing DL-dihydrosphingosine analog were separated on the same column through a modified water/ACN gradient starting from 95% to 56% water over 5 min, from 56% to 52% water over 2 min, followed by a linear gradient from 52% to 48% water over 1 min. The mass spectrometer was operated in positive mode and operated using a protocol optimized for each compound. Briefly, the center mass for dodecylamine analog was 1,037.66 Daltons (Da) and the scan width was 0.3 Da. The center mass for DL-dihydrosphingosine analog was 1,153.75 Da and the scan width was 1.0 Da. Area of each compound was measured through manual integration using Xcalibur™ Software (Thermo Fisher Scientific). Native occidiofungin served as the internal standard for all analogs. The *R*^2^ values for each standard curve exceeded 0.98.

### Actin polymerization and depolymerization assay

Actin polymerization and depolymerization assays were performed using the Actin polymerization biochem kit (# BK003; Cytoskeleton, Inc. Denver, CO) following the manufacturer’s instructions with some modifications. Briefly, for the polymerization assay, G-actin stock solution was made at 0.2 mg/ml instead of 0.4 mg/ml. For the depolymerization assay, F-actin stock solution was diluted nine-fold instead of five-fold. Test compounds including phalloidin, DL-dihydrosphingosine analog, and native occidiofungin were tested at a final concentration of 5 μM. The experiments were done in duplicate.

## Results

### Synthesis of novel occidiofungin analogs

**Figure 3:**
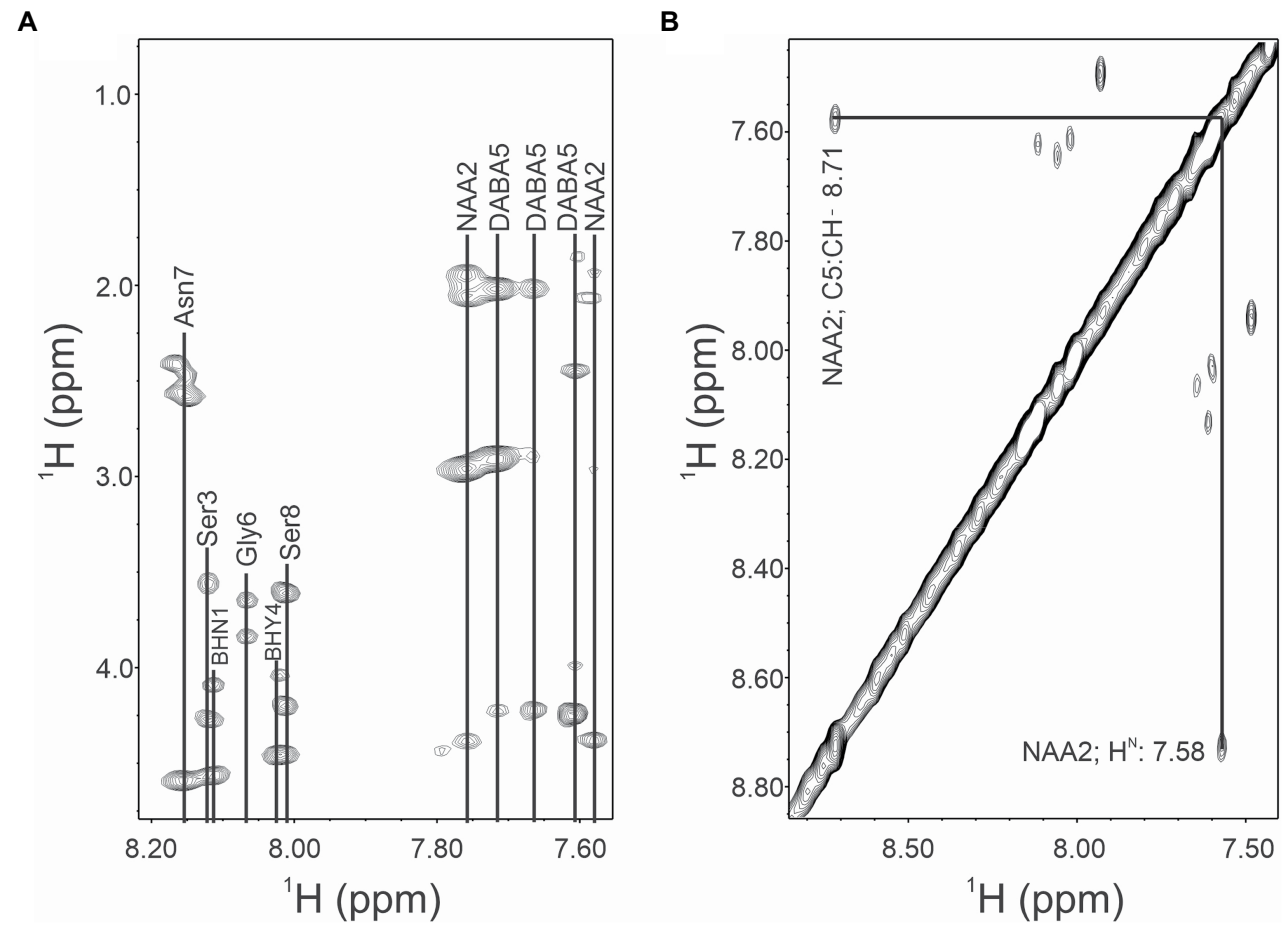
TOCSY and NOESY NMR spectra of occidiofungin. **(A)**. TOCSY spin system correlations of the aldehyde occidiofungin product. Fingerprint region (NH correlations), alpha to side chain correlations and side chain correlations are shown. Abbreviation are: diamino butyric acid 5 (DABA5), novel amino acid 2 (NAA2), beta-hydroxy-asparagine 1 (BHN1), and beta-hydroxy-tyrosine 4 (BHY4). **(B)**. NOESY spin system correlations. The expansion shows the intra-residue NOE interaction of the aldehyde proton of NAA2 to the amide proton of the NAA2.

The removal of the aliphatic chain resulted in a highly polar compound (Figure 1), which was isolated by HPLC under isocratic conditions. Oxidative cleavage of the vicinal diols proceeded efficiently, and occidiofungin was completely converted to the aldehyde product (Figures 1B, 2A). The isolated aldehyde product had the expected mass of 868.34 Da. Further, NMR analyses of the isolated product confirmed the isolation of the cyclic peptide with the aldehyde on carbon five C(5) of the NAA2 residue. The individual amino acid spin systems in the 2D TOCSY spectra are shown in Figure 3A and the chemical shifts for the aldehyde product are provided in Table 1. The through-space proton interaction between the aldehyde proton and the amide proton of the novel amino acid were observed in the 2D NOESY spectra (Figure 3B), supporting the proton chemical shift assignments of the aldehyde product. The aldehyde occidiofungin product was subsequently used in reductive amination reactions affording the synthesis of new analogs of occidiofungin (Abdel-Magid et al., 1996).

**Table 1:** NMR chemical shifts for aldehyde analog of occidiofungin.

Amino acid	H^N^	H^α^	H^β^	Other protons
BHN1	8.11	4.57	4.09	β-OH: 5.68, γ-NH2: 7.20,6.70
NAA2	7.76 7.58			C2:CH2–2.08&1.92, C3:CH- 4.39, C4:CH2–2.96, C5:CH- 8.71
Ser3	8.12	4.27	3.56	β-OH: 4.99
BHY4	8.02	4.45	4.04	β-OH: 5.71, OH – 9.28, C2&C6:CH – 7.16, C3&C5:CH – 6.70
DABA5	7.61	4.23	2.02	γ-H: 2.91, NH2: 7.71 γ-H: 2.87, NH2: 7.66
Gly6	8.07	3.82, 3.64		
Asn7	8.16	4.59	2.58, 2.47	γ-NH2: 7.44,6.97
Ser8	8.01	4.20	3.61	β-OH: 5.08

Proton chemical shift values were from a TOCSY and NOESY experiments.

**Figure 4:**
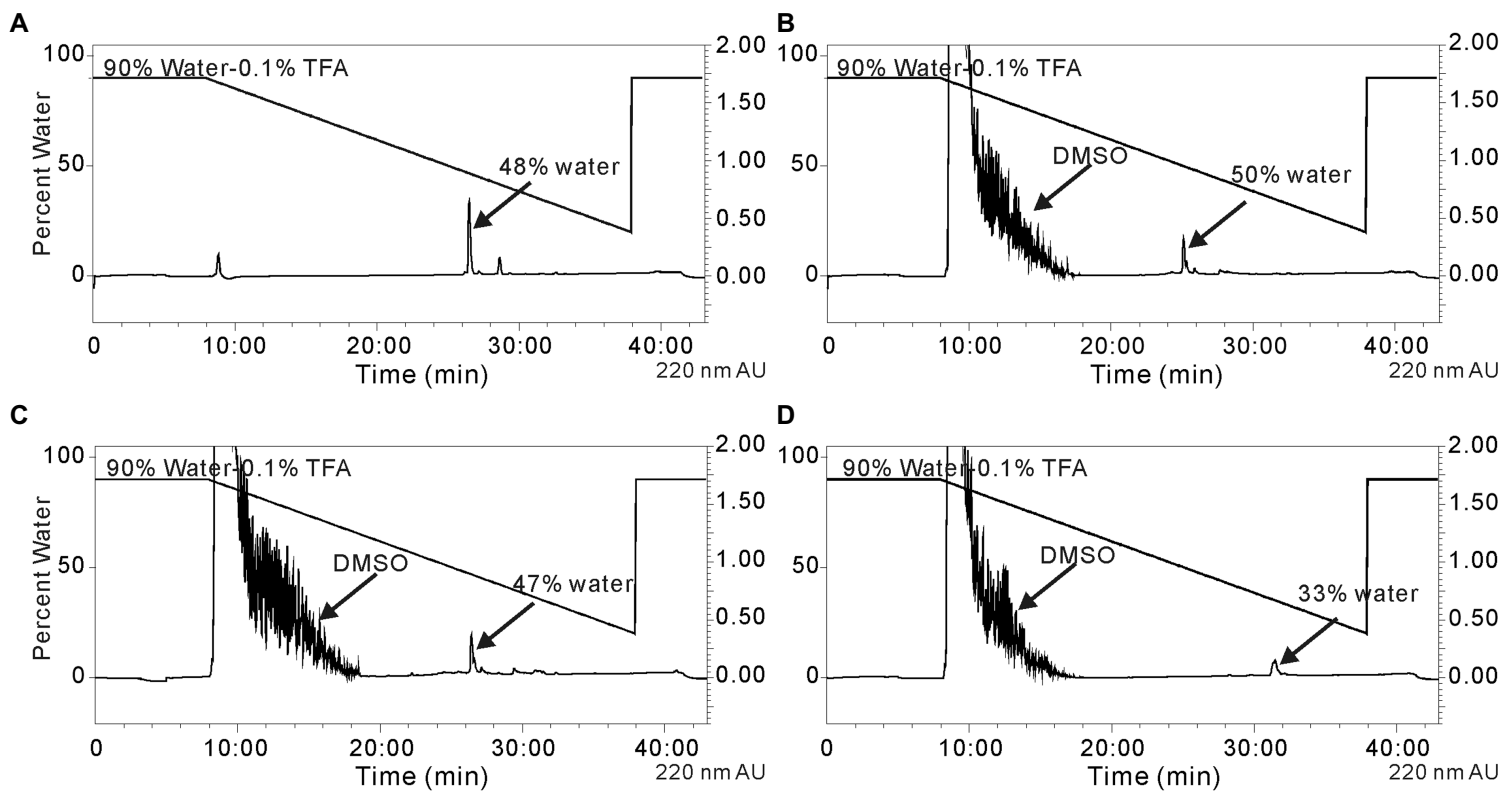
Isolation of the reductive amination products by HPLC. **(A)** native occidiofungin, **(B)** undecylamine analog following the reaction of the aldehyde analog with undecylamine, **(C)** dodecylamine analog following the reaction of the aldehyde analog with dodecylamine, and **(D)** the DL-dihydrosphingosine analog following the reaction of the aldehyde analog with d DL-dihydrosphingosine. The peak indicated by arrow is the desired product. The percentage of water at which the product being eluted from the column is labeled. Black lines represent the gradient of water with 0.1%TFA to acetonitrile. Absorbance (AU) was monitored at 220 nm. The noise in the beginning of the runs is due to DMSO in the reaction mixture.

Reductive amination reactions using three commercially available long chain alkyl amines and the aldehyde occidiofungin products were performed (Figure 2B). The oxidative cleavage of the diol in the NAA2 resulted in a loss of 13 carbons in the aliphatic chain. Since the xylose itself is not required for the antifungal activity (Chen et al., 2013), undecylamine and dodecylamine were used to restore the length of the aliphatic chain. A secondary amine is incorporated at the carbon six C(6) position and an aliphatic chain of 11 and 12 carbons were introduced for the undecylamine and dodecylamine products, respectively (Figure 2C). The alkyl amine DL-dihydrosphingosine was chosen because a hydroxyl group would be reintroduced into the side chain near the normally occurring positions found within the native product (Figure 2C). The unoptimized reactions were very efficient. The yields of the semisynthetic undecylamine, dodecylamine, and DL-dihydrosphingosine analogs were 39%, 54%, and 26%, respectively (Table 2), and it is likely that these yields can be improved by further optimizing the reaction methods. The percentage of water for the HPLC retention times was 48% for native occidiofungin, 50% for undecylamine analog, 47% for dodecylamine analog, 33% for (D) DL-dihydrosphingosine analog, and 46% for the xylose free occidiofungin (Figure 4). The final undecylamine, dodecylamine, and DL-dihydrosphingosine occidiofungin products were confirmed by high-resolution mass spectrometry (HRMS). For all the analogs, the HRMS data for each product was within 2 ppm of the expected masses (Table 2). Additional analogs can be rapidly synthesized by this approach and the structural diversity of analogs made can be expanded by the synthesis of novel amino lipid compounds that are not readily available.

**Table 2:** Summary of reactions.

Starting material	Amine	Yield (%)	Molecular weight (Da)		
			Expected mass	Measured mass	Mass error (ppm)
Aldehyde analog	Undecylamine	39	1023.5469	1023.5481	1.1723
Aldehyde analog	Dodecylamine	54	1037.5626	1037.5644	1.7348
Aldehyde analog	DL-dihydrosphingosine	26	1153.6463	1153.6476	1.1268

The starting materials and yield of each reaction are shown. The mass of the expected products was confirmed by high-resolution mass spectrometry. Mass error in parts per million (ppm) for each product was calculated based on the expected and measured mass. The yield of the reductive amination reactions was based on the HPLC peak areas of the starting material and product.

### Bioactivities of occidiofungin analogs

The inhibitory activities of the aldehyde, undecylamine, dodecylamine, DL-dihydrosphingosine analogs of occidiofungin were determined against *S. cerevisiae*, *Candida albicans*, and *Candida glabrata* (Table 3). The aldehyde, undecylamine, and dodecylamine analogs were inactive at concentrations 64-fold higher than the inhibitory concentration of native occidiofungin against the *Candida* species tested and *S. cerevisiae* strain tested (Table 3). The undecylamine and dodecylamine analogs have a similar aliphatic carbon length as native occidiofungin. Further, these analogs have similar polarity as native occidiofungin, as was observed by the HPLC retention times. The major structural differences to native compound are the conversion of a C(5)-C(6) to C(5)-N(6) bond in the side chain of NAA2, the removal of the vicinal diol group, and the loss of a xylose sugar. DL-dihydrosphingosine analog was synthesized to test whether the introduction of a hydroxyl group near C(6) position could restore activity. DL-dihydrosphingosine analog has one branched alcohol on C(7) and a hydroxyl on C(8) positions normally found within native occidiofungin. Further, the aliphatic chain of DL-dihydrosphingosine analog is five carbons longer than the native compound. The DL-dihydrosphingosine partially restored antifungal activity as observed by a low micromolar inhibitory activity against *S. cerevisiae*, *C. albicans*, and *C. glabrata*; the MICs were 2, 16, and 8 μg/ml, respectively. The loss of activity, or the reduction of activity, of the semisynthetic analogs of occidiofungin could be attributed to a reduction in their ability to penetrate the plasma membrane to reach cellular target (binding to cell envelope or inability to cross plasma membrane) or a loss in affinity to actin (the cellular target of occidiofungin).

**Table 3:** MIC (μg/ml) of occidiofungin analogs against *Saccharomyces cerevisiae* DGY6 haploid BY4741, *Candida albicans* ATCC 3147, and *Candida glabrata* ATCC 2001.

Product	*S. cerevisiae* DGY6	*C. albicans* ATCC 3147	*C. glabrata* ATCC 2001
Native occidiofungin	0.0625	0.5	0.5
Xylose-free occidiofungin	0.0625	0.5	-
Occidiofungin aldehyde	>4	> 8	>8
Undecylamine	>4	>32	>8
Dodecylamine	>4	>32	>8
DL-dihydrosphingosine	2	16	8

### Competitive binding assays

**Figure 5:**
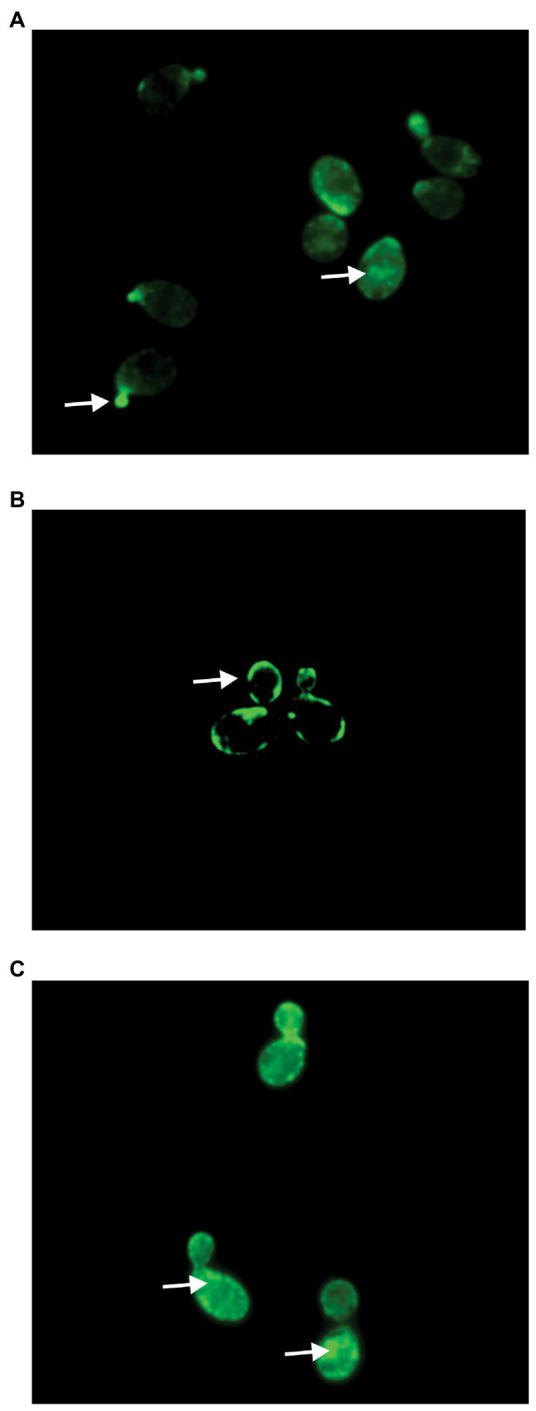
Fluorescent microscopy of *Saccharomyces cerevisiae* cells treated with **(A)** alkyne occidiofungin for 30 min, **(B)** native occidiofungin for 5 min followed by alkyne occidiofungin for 30 min (image intensity adjusted to 30% given increase in nonspecific binding to cell envelope), **(C)** dodecylamine analog for 5 min followed by alkyne occidiofungin for 30 min. Green fluorescence indicates location of alkyne occidiofungin. White arrows demarcate specific locations of alkyne-OCF at bud tips, intracellular inclusions or at the cell envelope.

Previous studies by our lab demonstrated that the modified alkyne derivative of occidiofungin (alkyne-OCF) used for fluorescent labeling studies is found intracellularly in *S. cerevisiae* cells at 30 min post incubation where it is associated with large intracellular inclusions (Ravichandran et al., 2019). Similarly in this study, accumulation of alkyne-OCF was found to be localized within the cell and at bud tips (Figure 5A). Previously, native occidiofungin was shown to prevent uptake of the alkyne-OCF (Ravichandran et al., 2019). *Saccharomyces cerevisiae* cells were pretreated with native occidiofungin for 5 min, followed by treatment with alkyne-OCF. Pretreatment with native occidiofungin prevented internalization of alkyne-OCF (Figure 5B). There was a concomitant increase in fluorescence at the surface of the cell envelope. In this study, we wanted to determine whether the dodecylamine analog could prevent the uptake of the alkyne-OCF in a similar competition bioactivity assay. Pretreatment with dodecylamine analog of occidiofungin did not interfere with the internalization of the alkyne-OCF and showed similar localization patterns as treatment with solely alkyne-OCF (Figure 5C). Pretreatment with native occidiofungin does prevent alkyne-OCF from entering the cell, while the dodecylamine analog did not compete with alkyne-OCF for uptake into the cell.

### Actin binding properties of occidiofungin analogs

**Figure 6:**
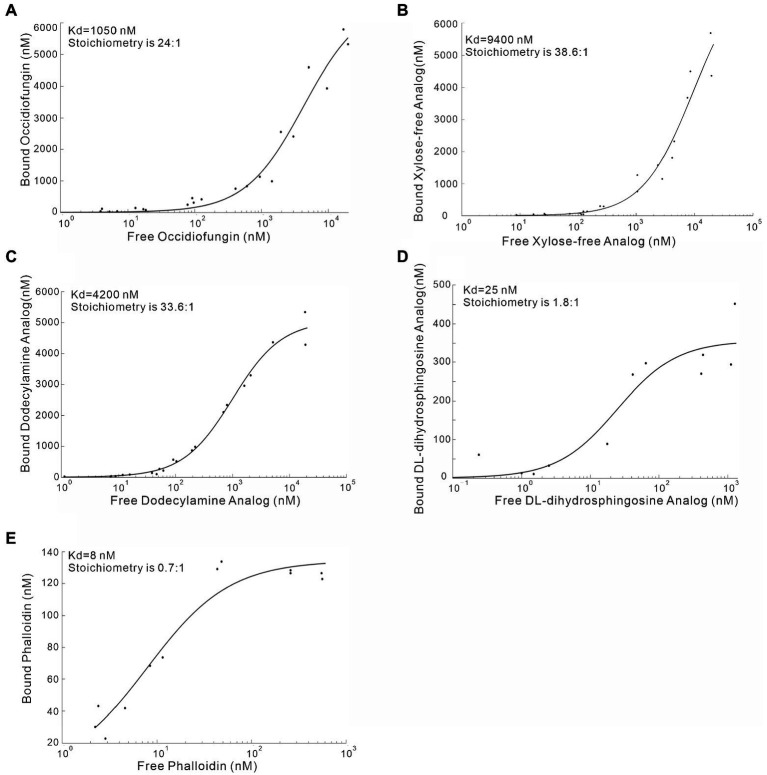
Co-sedimentation assay demonstrating the binding of occidiofungin analogs to actin. **(A)** Binding curve of native occidiofungin to actin (Kd = 1,000 nM; the stoichiometry [ligand: protein] is 25: 1. **(B)** Binding curve of xylose free occidiofungin to actin (Kd = 9,400 nM; the stoichiometry [ligand: protein] is 38.6: 1. **(C)** Binding curve of the dodecylamine analog to actin (Kd = 4,200 nM; the stoichiometry [ligand: protein] is 33.6: 1. **(D)** Binding curve of the DL-dihydrosphingosine analog to actin (Kd = 25 nM; the stoichiometry [ligand: protein] is 1.8: 1. **(E)** Binding curve of phalloidin to actin (Kd = 8 nM; the stoichiometry [ligand: protein] is 0.7: 1. The graph is plotted between the amount of free compound obtained in the supernatant of the co-sedimentation assay and the amount of bound compound obtained from the actin pellet. Data for native occidiofungin and phalloidin has been published previously (Ravichandran et al., 2019).

To characterize the F-actin binding properties of the aldehyde, dodecylamine and DL-dihydrosphingosine analogs of occidiofungin, a co-sedimentation assay was performed. In a previous study, we showed that native occidiofungin had a Kd value of 1,050 nM and a saturation of binding ratio of ~24:1 (ligand:actin monomer; Ravichandran et al., 2019). Further, we showed that phalloidin had an estimated Kd value of ~8 nM with a saturation of binding ratio of ~0.6:1 (ligand:actin monomer), which is in agreement with previously reported values (De La Cruz and Pollard, 1996). In this study, we evaluated the binding of the aldehyde analog, xylose free occidiofungin, dodecylamine analog, and the DL-dihydrosphingosine analog to F-actin. A dissociation constant for the aldehyde analog could not be determined given that it did not bind to F-actin in the co-sedimentation assay. The xylose free occidiofungin was determined to have a Kd value of 9,600 nM with a saturation of binding ratio of ~38.6:1 (ligand:actin monomer; Figure 6). The dodecylamine analog was determined to have a Kd value of 4,200 nM with a saturation of binding ratio of ~34:1 (ligand:actin monomer). Interestingly, the DL-dihydrosphingosine analog had a Kd value of 25 nM with a saturation of binding ratio of ~1.8:1 (ligand:actin monomer). The xylose free occidiofungin and the dodecylamine analog both had a higher saturation of binding to actin than native occidiofungin with approximately a nine-fold and four-fold lower binding affinity to actin, respectively. The DL-dihydrosphingosine analog showed an actin binding property closer to phalloidin’s and had a 13.5-fold lower saturation of binding ratio than native occidiofungin. The decrease in dissociation constant for the DL-dihydrosphingosine analog is likely attributed to the low saturation of binding compared to native occidiofungin and not an increase in affinity to actin.

**Figure 7:**
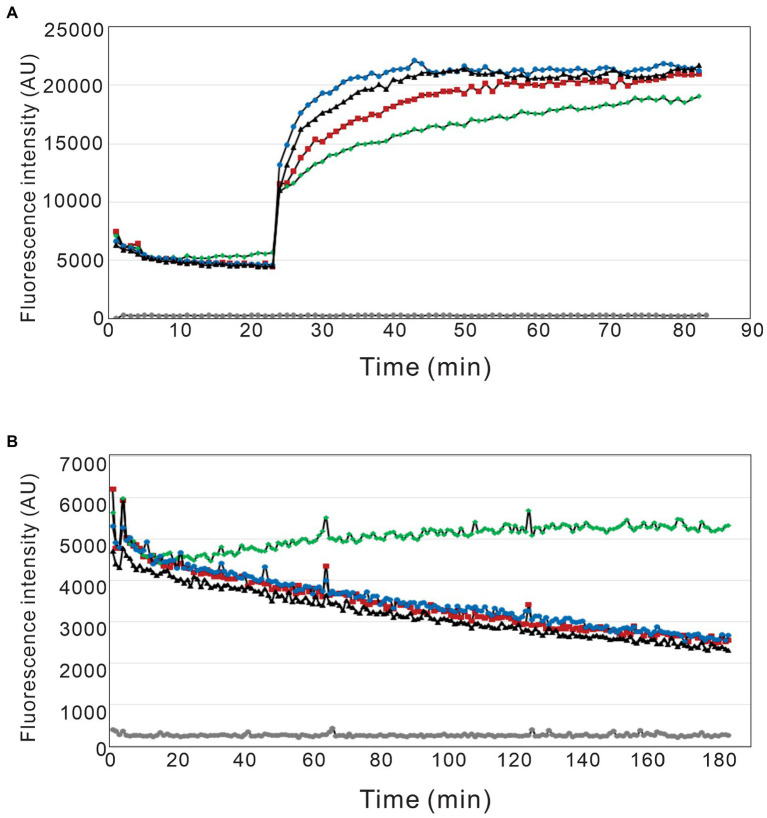
Pyrene labeled actin polymerization and depolymerization assay. The effect of phalloidin, native occidiofungin, and the DL-dihydrosphingosine analog on actin **(A)** polymerization and **(B)** depolymerization assays. (grey ●) represents buffer baseline control; (red ■) represents no drug control; (green ◆) represents phalloidin treatment; (blue ●) represents native occidiofungin treatment; (black▲) represents DL-dihydrosphingosine analog treatment. The fluorescent data is measured in terms of arbitrary units (AU).

Given that the actin binding properties of the DL-dihydrosphingosine analog were different from native occidiofungin, the analog was tested to determine whether its mechanism of actin binding was different than native occidiofungin. Native occidiofungin does not interfere with actin polymerization or depolymerization, but rather interferes with actin cable formation triggering ROS accumulation and apoptotic cell death (Ravichandran et al., 2019). The molar ratio of occidiofungin, analogs of occidiofungin, or phalloidin to actin in the polymerization and depolymerization assay was close to 1:1. The pyrene fluorescence readings approximately 1 hour after incubation in the polymerization buffer plateaued at around 19,000 for phalloidin and 21,000 AU for the DL-dihydrosphingosine analog and the native occidiofungin (Figure 7). Following incubation in the depolymerization buffer, actin filaments incubated with phalloidin had pyrene fluorescent readings above 5,000 AU, while actin filaments incubated with DL-dihydrosphingosine analog and the native occidiofungin had similar readings as no drug control at ~2,500 AU. Phalloidin completely blocked the depolymerization of F-actin, which is in agreement with previous studies (Coluccio and Tilney, 1984; Cooper, 1987). The DL-dihydrosphingosine analog did not interfere with polymerization or depolymerization of actin, similar to what was observed with native compound (Figure 7). The lack of any interference with the polymerization or depolymerization of actin by the DL-dihydrosphingosine analog supports the assumption that the DL-dihydrosphingosine analog and native occidiofungin have the same binding region on actin.

## Discussion

In this study, a semisynthetic method to generate novel occidiofungin analogs has been developed. As proof of principle, three novel analogs of occidiofungin with varying amino alkyl groups were synthesized; these analogs were studied to determine their antifungal activity and actin binding properties. Oxidative cleavage of the diol group present in the aliphatic chain of NAA2 followed by reductive aminations of alkyl amines restored the aliphatic structure of the native compound. The results showed that the fatty acid moiety in native occidiofungin plays an important role with regards to its antifungal activity and actin binding properties. The novel DL-dihydrosphingosine analog of occidiofungin retained low micromolar antifungal activity. Despite having reduced inhibitory activity compared to the native compound, the findings support future studies aimed at expanding the library of semisynthetic compounds that can be synthesized using this methodology. The use of DL-dihydrosphingosine analog of occidiofungin does show that bioactivity can be restored by introducing this alkyl side chain containing alcohol moieties. Further, the analogs evaluated in this study have improved our current understanding of the bioactivity of occidiofungin. Methods used to synthesize analogs by this route can be used to probe fundamental questions on cellular entry of this unique antifungal agent and its mechanics of action.

While the mechanism for cellular entry for occidiofungin still remains unclear, occidiofungin needs to enter the cell to reach its cellular target actin. The undecylamine and dodecylamine analogs are both completely inactive at a concentration that was 64-fold higher than native compound against *S. cerevisiae*, while the dodecylamine analog only had a four-fold increase in the dissociation constant for actin. The lack of activity of undecylamine and dodecylamine analogs suggests that the hydroxyl groups on the side chain of NAA2 may be essential for the uptake of occidiofungin into the cells. This assumption is further supported by the observation of xylose free occidiofungin having a similar inhibitory activity as native occidiofungin (Table 3), while the xylose free occidiofungin had a higher dissociation constant than the dodecylamine analog (Figure 6B). Furthermore, the dodecylamine analog was capable of binding to F-actin in a similar manner as native occidiofungin (Figure 6C). Given that the inhibitory activity of the DL-dihydrosphingosine analog can be partially restored, we hypothesize that the introduction of the hydroxyl groups near the base of the lipid moiety may aid in cellular entry and enable the analog to reach the actin target. The acyl lipid analogs synthesized in this study may be useful in future studies aimed at furthering our understanding of the mechanism of cellular entry of occidiofungin in fungi.

While the mechanism used by occidiofungin to cross the plasma membrane to enter the cell is still unknown, the engineered semisynthetic analogs are capable of binding to actin similar to native occidiofungin. From this study, it is likely that the NAA2 and the diol region in particular is a crucial structural region for permitting cellular entry of occidiofungin. The intracellular localization pattern of occidiofungin is disrupted when *S. cerevisiae* cells are pretreated with native occidiofungin (Figure 5B). Pretreatment with native occidiofungin resulted in the accumulation of alkyne occidiofungin at the cell envelope. Pretreatment with native occidiofungin likely alters the cell envelop composition leading to nonspecific accumulation of the alkyne-OCF at the cell surface. Native occidiofungin may trigger blebbing or other biological processes that result in accumulation of actin at the cell surface, hence could be responsible for the strong fluorescent signal observed (Ren et al., 2021). Since pretreatment with dodecylamine analog did not interfere with alkyne-OCF internalization (Figure 5C), the dodecylamine analog is likely inactive given that it cannot enter the cell. Additional studies toward engineering analogs that can cross the plasma membrane or the development of novel formulations to promote cellular uptake are needed.

It is well known that exogenous fatty acids can be readily taken up by yeast (Tehlivets et al., 1771; Færgeman et al., 1997). For instance, Fat1p, a homolog to the murine fatty acid transport protein, has been demonstrated to be a long-chain fatty acid transport protein on the membrane of *S. cerevisiae* (Færgeman et al., 1997). Whether a fatty acid transport protein is involved, or other mechanisms are involved will need further investigation. Liposomes are widely used as drug delivery vehicles to transfer chemotherapeutic agents into target cells (Un et al., 2012). In future studies, the undecylamine and dodecylamine analogs will be encapsulated in liposomes and their inhibitory activities will be reevaluated. It may be possible to restore inhibitory activity by promoting the permeabilization of the membrane and cellular uptake of the occidiofungin analogs. Further, the acyl lipid analogs can be modified at the 2,4-diaminobutyric acid position in a similar manner that has been performed for native occidiofungin (Ravichandran et al., 2019). Alkyne labeled undecylamine and dodecylamine analogs will enable fluorescent microscopy studies aimed at determining whether the analogs can penetrate the cell membrane.

The low binding constant and saturation stoichiometry of the DL-dihydrosphingosine analog is an interesting observation. The high saturation of occidiofungin to actin (24:1) might be attributed to the self-assembly mechanism of native occidiofungin monomers following its binding to F-actin. This is not uncommon activity for lipopeptides and the formation of self-assembled complexes was observed with several lipopeptide antibiotics (Hamley, 2015). The polarity of DL-dihydrosphingosine analog is much less than the native compound (eluted at 33% versus 47% water on HPLC (Figure 4D)), which might contribute to the loss of the self-assembly mechanism, in the sense that amphiphilicity leads to self-assembly at high concentrations for peptide amphiphiles (Hamley, 2011). Another possible explanation for the loss in self-assembly properties is the presence of the branched alcohol at C(7) on the DL-dihydrosphingosine. The relationship between self-assembly and bioactivity is complicated and remains unclear (Hamley, 2015) and the self-assembly property of occidiofungin will need further investigation.

The DL-dihydrosphingosine analog, which lacks the self-assembly activity, will be an ideal candidate to study the primary binding site of occidiofungin on F-actin and further the understanding of its biological activity. The DL-dihydrosphingosine analog had a Kd value of 25 nM and had a stoichiometry closer to a 1:1 (ligand:actin) binding. It is unlikely that the DL-dihydrosphingosine analog has a higher affinity for actin. The lower Kd value likely represents the microscopic dissociation constant (which captures the affinity of one occidiofungin binding to actin) versus the macroscopic Kd value captured by the native compound (24:1 ligand:actin), as well as the values reported for the xylose-free analog and the dodecylamine analog.

Current antifungal treatments are challenged with wide-spread resistance, limited spectrum of activity, and toxicity associated with administration. Occidiofungin has a unique mechanism of action, wide spectrum of activity, and minimal toxicity in a mouse model. Different from antifungal compounds jasplakinolide and halichondramide which inhibit polymerization or depolymerization of actin filaments and result in severe toxicities (Bubb et al., 1994; Chung et al., 2011), occidiofungin has a subtle effect on actin dynamics that triggers apoptotic cell death. Due to its unique mechanism of action, occidiofungin has sub-micromolar activity against azole and echinocandin resistant strains of fungi (Ravichandran et al., 2019). The described chemical approach to produce semisynthetic analogs has the ability to lend itself to rapidly create novel analogs of occidiofungin. Even though the activity of the DL-dihydrosphingosine analog was 16 to 32-fold lower than the native compound, the activity was still within the low micromolar range. Future work will focus on the synthesis and characterization of analogs with varying physical and chemical properties. The analogs with improved or similar antifungal activities as the native compound will be studied for improvements in toxicity and efficacy. The study has also demonstrated that the diol group on NAA2 plays a key role in its bioactivity and is structurally important for cellular uptake. Further, the study has also opened up a number of new questions about mechanism for cellular entry and the specific site for actin binding. The findings within this study lay a solid foundation that will help address these questions.

## Data availability statement

The raw data supporting the conclusions of this article will be made available by the authors, without unduereservation.

## Author contributions

MG, NH, RO, and LS either helped to plan or perform the experiments. S-EL, SL, and RS helped to analyze the data. RO and LS supervised the study and designed the experiments. MG, NH, and LS drafted the manuscript. All authorshave read and agreed to the published version of the manuscript.

## Funding

The NMR experiments were supported by the NIH grants NIH-NIAID R42AI131792, 1P30 CA-13148 and 1S10 RR022994-01A1. The research was funded by National Institute of Health R41AI131792–01 and 2R42AI131792-02A1.

## Conflict of interest

LS and S-EL are board members of Sano Chemicals Inc. Sano Chemicals is actively developing occidiofungin for the treatment of serious fungal infections.

The remaining authors declare that the research was conducted in the absence of any commercial or financial relationships that could be construed as a potential conflict of interest.

## Publisher’s note

All claims expressed in this article are solely those of the authors and do not necessarily represent those of their affiliated organizations, or those of the publisher, the editors and the reviewers. Any product that may be evaluated in this article, or claim that may be made by its manufacturer, is not guaranteed or endorsed by the publisher.
